# Experiences of cervical cancer patients in rural Ghana: An exploratory study

**DOI:** 10.1371/journal.pone.0185829

**Published:** 2017-10-11

**Authors:** Charity Binka, David Teye Doku, Kofi Awusabo-Asare

**Affiliations:** 1 School of Public Service and Governance, Ghana Institute of Management and Public Administration, Accra, Ghana; 2 School of Health Sciences, University of Tampere, Tampere, Finland; 3 Department of Population and Health, University of Cape Coast, Cape Coast, Ghana; Rudjer Boskovic Institute, CROATIA

## Abstract

Even though cervical cancer is quite a prevalent disease in Ghana, there is hardly any study on this disease. This paper sought to explore the experiences of cervical cancer patients living with the disease with emphasis on their knowledge about the disease before and after the diagnosis. Qualitative data were collected through in-depth interviews with cervical cancer patients undergoing treatment in a specialised cancer treatment health facility in rural Ghana.

Cervical cancer patients had inadequate knowledge about the disease, its symptoms, risk factors, treatment and prevention prior to being diagnosed of the disease. These patients were diagnosed late because they usually sought treatment elsewhere before reporting to health facilities. They experienced physical, psychological, economic and social disruptions in their daily lives, which affected their quality of life. It is evident that lack of knowledge about cervical cancer constitutes a threat to its prevention and treatment. Intensive health education through the mass media and community health promotion outreaches can be a sure way of creating adequate knowledge about cervical cancer in Ghana. Treatment and care for cervical cancer patients should incorporate counselling sessions, which should take into consideration the different levels of disruption the women experience and the implications for their wellbeing and management of the condition.

## Introduction

Cervical cancer is the third most commonly diagnosed cancer worldwide and the fourth leading cause of cancer deaths among women globally [1]. The disease is estimated to cause about 266,000 deaths annually worldwide [2]. Over the past decades, the incidence of cervical cancer has however reduced by a third and mortality by half. It has been shown that the cervical screening programme is associated with the improvement in cervical cancer treatment rates [3].

In Ghana, it is estimated that about 3,052 new cervical cancer cases were recorded in 2012, representing 32.7 per cent of the total cancer cases among women in the country [2]. Cervical cancer is believed to be highly preventable with the use of cervical cancer screening tools [4]. In the early stage, cervical cancer can easily be treated. In its advanced stage, the treatment of the disease could be very challenging or impossible [4]. Owing to low awareness of cervical cancer in Ghana, over 90 per cent of the cases presented in health facilities tend to be at the advanced stage where the disease has already spread [5]. Consequently, cervical cancer incidence and mortality rates in Ghana become one of the highest in the world [6].

A number of studies in Ghana on cervical cancer did not focus on those suffering from the disease. Rather, the studies were more on the prevalence of cervical cancer and screening. None have investigated the phenomenon among patients. Previous studies emphasized on inadequate knowledge of the disease among their respondents [7–10]. The low level of knowledge about the disease results in low uptake of screening and treatment [11]. Respondents were found to lack knowledge on the risk factors, screening services, symptoms and treatment options of the disease [8,9,12]. The present study among cervical patients therefore sought to explore their experiences as well as patients’ knowledge about the disease prior to diagnosis. Understanding cervical patients’ experiences will help in determining effective interventions that can improve care for cervical cancer patients. The experience of the cancer patients was guided by the concept of biographical disruption [13]. According to Bury [13], chronic diseases bring about disruption to a patient’s life trajectory and biography. In other words, chronic diseases create discontinuity in the life of patients. He argues that the lives of people living with a chronic illness are disrupted and full of uncertainties. The situation makes them focus on their bodily sufferings, resulting in ruptures between body, self and society [13, 14].

Bury [13] therefore argues that the onset of a chronic disease brings pain, suffering and death to the fore, things that are normally seen as distant possibilities in ones’ life or perceived as the problem of other people. As a result of this suffering, people’s life trajectories are disrupted. The concept of biographical disruption has been used as a conceptual framework in studying the experiences of persons living with a number of chronic illnesses such as AIDS [15], stroke [16], and lung cancer [17]. From these studies, chronic diseases have been found to disrupt the body, social identity, social relationships, career decision and economic condition of participants [16, 18]. Based on the previous studies, the biographical disruption concept was used to guide this paper.

## Materials and methods

### Study setting and design

This study was conducted at the Battor Catholic Hospital in the North Tongu District of the Volta Region in Ghana. It is the only health facility that provides cervical cancer screening and treatment in a rural environment in the country. A qualitative research method was adopted for this study. The qualitative approach was used because it helped to explore and understand the experiences of the cervical cancer patients.

### Study population

The study population of the study comprised all women who attended the gynaecology unit of the health facility and had been diagnosed with cervical cancer and had survived the disease or at its early stage. The respondents were recruited based on the following criteria: whether they were diagnosed with cervical cancer by the gynaecology unit of the health facility; women who were 30 years and above, and willing to participate in the study. The age range was in line with the cervical cancer guideline which recommends that every woman from age 30 to 49 should at a minimum have cervical cancer screening at least once in her life time [2]. It has also been established that the prevalence of Human Papilloma Virus (HPV), which is recognised as the major risk factor for the disease, is highest among women in their reproductive age and beyond, followed by post-menopausal women [11]. The respondents included those who were in the early stage of the disease and those undergoing surgery or treatment. Patients who were in life-threatening conditions and had undergone removal of the cervix (total hysterectomy) at the time of the study were, however, excluded from the study.

### Sampling procedure

The sampling technique adopted was purposive. Thus, only women who had survived cervical cancer or were living with the early stage of the disease were considered for the purpose of the study. In recruiting respondents, the records of patients were reviewed at the cancer registry to identify patients who had been diagnosed with the disease. In total, 60 patients were identified from the hospital register at the time of the study. Afterward, phone calls were made to patients to seek their participation in the study. Many could not be reached because they were either dead or the telephone numbers recorded in the register were inaccurate. Some of the patients who were contacted also declined to participate in the study. Consequently, only 15 women have agreed to participate in the study. These women were all included in the study and their responses provided evidence to support the findings of this study.

### Data collection

An in-depth interview guide was developed to collect data for the study. This comprised issues on background characteristics of respondents, personal coping strategies, forms of support and health seeking behaviour of the respondents. The respondents were interviewed in three Ghanaian languages, namely, Ewe, Twi and Dangme. Three female research assistants who were fluent in the three languages were recruited and trained on the contents of the data collection instrument and how to effectively conduct the interviews to ensure data quality and without compromising ethical issues.

To ensure the accuracy of data collected, the researchers and field assistants immediately conducted checks with the respondents after the interviews to verify their trustworthiness. The interviews were also peer reviewed by the research team.

### Data processing and analysis

The interviews were tape-recorded and transcribed in English by research assistants who were native speakers of the local languages and fluent in the English language. The transcripts were then given to language experts in the three local languages to review and revise the content to ensure validity of findings. The transcribed interviews were coded and processed with the R software package for Qualitative Data Analysis (RQDA) (version R-3.2.2). A thematic approach to qualitative data analysis was therefore applied. This involved generating deductive codes that emerged from the interviews. The results were presented in quotations from the respondents to support the contextual analysis and provide an opportunity for discussion of the results of the study.

### Ethics approval and consent to participate

Institutional approval for the study was obtained from the Battor Catholic Hospital, while the Ghana Health Service Ethical Review Committee (GHS-ERC) granted clearance for the work. Respondents were made to sign a written informed consent to participate in the study. Additionally, respondents were assured of anonymity and confidentiality of information.

## Results

### Background characteristics of cervical cancer patients

The background characteristics of the 15 cervical cancer patients who participated in the study were summarised. Out of the 15 cervical cancer patients, six were aged 50–59 while four were aged 40–49 and 60 and above each. One participant had no formal education, four had primary school education and eight had secondary school education while two had tertiary education. Ten out of the 15 patients were married while the remaining five were divorced, separated or widowed. Also, ten were self-employed while the rest were unemployed. Nine participants had from 4 to 6 children while three had 7 or more children. All the 15 participants were Christians. Among the patients, only one indulged in smoking tobacco while four reported taking alcohol.

### Knowledge about cervical cancer

Respondents were asked what they perceived as the causes of the cervical cancer disease. All the women interviewed (n = 15) did not know about cervical cancer prior to diagnosis and expressed surprise at the situation in which they found themselves. The themes explored on knowledge of the patients of cervical cancer are presented in Table 1 below.

**Table 1:** Knowledge of cervical cancer.

Issue	Explanation
Knowledge about existence of cervical cancer	All the patients did not know about the disease before diagnosis
Population at risk	13 of the patients did not know the population at risk
Causes	14 of the patients did not know the causes of the disease, only one mentioned HPV and indiscriminate sex.
Screening	None of the patients knew about screening options
Treatment	None of the patients knew about treatment options

Source: Fieldwork, 2014

The patients said the diagnosis came as a surprise to them as they had no prior knowledge about cervical cancer as captured in the responses below.

*“I did not even know anything about cancer*. *I knew it was only breast cancer that can affect women*. *I did not know there was anything like cervical cancer.”*

(Participant 2, 52 years).

*“I did not understand what was happening to me because I had no knowledge of the disease*. *I did not know the local name for this disease*”.

(Participant 13, 47 years).

The cervical cancer patients were not aware of the initial symptoms of the disease. For this reason, they could not identify the cause of their ailment until they were diagnosed. Two of the respondents expressed the frustration they experienced.

*“When I was bleeding*, *I did not know the kind of disease that was causing that to happen*. *I did not know what was wrong with me and what has caused this because I had stopped my period for about four years so when it started coming I was disturbed*. *I did not understand the reason why it had come to that until I got to the hospital and was told it was cancer”.*

(Participant 5, 50 years).

*“I did not understand why as a woman who had ceased childbearing*, *I am beginning to bleed again with vaginal discharge*. *I did not understand so I was always visiting the hospital with it”*.

(Participant 11, 55 years).

Respondents were asked what they perceived as the causes of the cervical cancer. Most of them (n = 13) reported that they did not know the causes of the disease prior to diagnosis. The “don’t know” responses were as result of genuine lack of knowledge about the condition and a conflict between their lifestyle and the causes of the diseases they have learnt. Two of the participants mentioned that it was after diagnosis that they realised that the disease could be caused by indiscriminate sex. They therefore explained that indiscriminate sex increases the risk of contracting the HPV, which is the main cause of cervical cancer:

“*After I was diagnosed*, *whenever there is any cervical cancer programme I listened*, *so I got to know that the disease is caused by indiscriminate sex and it is a virus.”*

(Participant 6, 63 years).

The cervical cancer patients got to know about some of the screening and treatment options for the disease after they were diagnosed. They got to know about the treatment options through the health personnel. The patients mentioned lying in a “machine”—CT scan, surgery. They did now other forms of cervical cancer treatment. Their knowledge about screening centres was limited as their focus was more on treating their condition.

*“When I came*, *the doctor had referred me to a certain hospital*. *He handed a letter to me to be sent to a certain hospital in Accra where I would be treated and that was where the results proved that it was cancer”.*

(Participant 3, 43 years).

*“Initially*, *when I came here*, *the doctor said because the cancer had advanced*, *I should go to a machine at Korle Bu Hospital*. *So when he said that*, *I agreed but I told him I was scared of the operation*, *so I would prefer to go and lie on the machine.”*

(Participant 5, 50 years).

A few of the respondents also mentioned some specific treatment options that they went through. These treatment options that were available included surgery and radiotherapy. The patients, however, only got to know about these treatment options during the treatment process when the doctors informed them. This is reflected in the following statements:

“The doctor suggested surgery as the other form of treatment for me and also radiotherapy”.

(Participant 7, 59 years).

*“I was told that it will be very difficult for them to operate*. *I was made to lie under a machine*. *I was then given injection and thereafter*, *I was operated upon and became okay.*

(Participant 8, 62 years).

### Illness experience and biographical disruption

The symptoms cervical cancer patients experienced signified the beginning of their biographical disruption. The disruptions were at four levels: physical, psychological, economic and social. These are discussed below.

#### Physical disruption

The symptoms of cervical cancer affected the bodies of these patients. Most of the women reported further disruptions to their physical body during treatment. The disruptions were in terms of body pains, cessation of sexual activity, loss of appetite, immobility and loss of sleep. The women experienced pains in their vagina, waist, and entire body. Table 2 represents the themes providing explanation to physical disruptions experienced by the cervical cancer patients.

**Table 2:** Physical disruption experienced by women.

Issue	Explanation
Body pains	Uncomfortable feeling in the lower abdomen
No sex	Pain during sex
Loss of appetite	Unwell, stressed, inability to eat
Immobility	Inability to walk around like any other normal human beings due to incessant bleeding
Loss of sleep	Inability to sleep due to bodily pains

Source: Fieldwork, 2014

For some of the women, the pains were so severe that they had to be regularly given injections to reduce it.

*“I felt some pains in my vagina and the doctor instructed that I should be given injection to reduce the pains*. *I was always in pain*, *my leg*, *everything*, *every part.”*

(Participant 11, 55 years).

Another physical disruption the women experienced was in their sexual life. Four of the women reported that they stopped engaging in sexual activity because they bled and felt pains during sex.

*“I bled from the vagina anytime I had sexual intercourse*. *So I decided not to get myself involved in any.”*

(Participant 4, 44 years).

Three women reported loss of appetite due to cervical cancer. Two of the women could not eat well while one had no interest in food. According to them, food smelt bad and was tasteless. The act of eating was seen as a burden. Another physical disruption women experienced was immobility. The women could not move around for fear of soiling themselves because of excessive bleeding. For example, three women said they were afraid to go out of their homes because they feared bleeding and soiling themselves in public. Some of the women could also not walk because they felt pains in their legs and body. According to seven of the women the drugs prescribed to them made them dizzy and prevented them from moving around.

*“I felt pains in my legs*. *I could not walk*. *The drugs given to me at Korle-Bu [one of the teaching hospitals] also weakened me and I could not do anything.”*

(Participant 8, 62 years).

The women also reported loss of sleep as disruption they experienced. For some of the women they could not sleep and others had to wake up intermittently to change their sanitary towels.

The physical disruption women experienced spanned across symptomatic, diagnosis, treatment and post treatment periods.

#### Psychological disruption

The women experienced psychological disruption in four phases: when they saw the disease symptoms, when they received diagnosis, during treatment and after treatment. These disruptions were clustered into three areas; namely, negative emotions, fear of death and anxiety. According to three patients, when they noticed the symptoms of the disease, they became sad and fearful. They were afraid of the blood they constantly saw in their underwear and wondered what could have been happening to them.

Some of the women also felt sad that something as embarrassing as that was happening to them. Table 3 represents the themes of the psychological disruptions experienced by the women.

**Table 3:** Psychological disruption experienced by cervical cancer patients.

Issue	Explanation
Negative emotions	Sadness, self-disgust, worry, desperation, bad feeling, confusion, loss of hope, isolated, tears and trauma.
Fear	Fear of the future, fear of death, fear of the unknown, treatment efficacy, fear of cost of treatment, fear of recurrence.
Thinking	Thinking about life after treatment

Source: Fieldwork, 2014

The patients experienced a lot of psychological disruption when they were first diagnosed of the disease. The women reported worry, anguish, desperation and loss of hope. For some of these women, they knew that cervical cancer was a deadly disease, and thought they were going to die immediately. One woman experienced psychological disruption because she was hoping that her condition was fibroid but not cervical cancer.

*“The day I got to know I had cervical cancer*, *I was devastated*. *But I gathered courage and raised my two hands and made a passionate request to save me so that I could continue doing my work*. *I prayed in my head but I raised my hand in front of the doctor.”*

(Participant 6, 63 years).

*“I felt sad*, *because of the cancer*. *I thought it was fibroid at first.”*

(Participant 9, 49 years).

According to some, they spent a lot of time thinking about the disease after they had been diagnosed. Their thoughts were focused on why such a thing had happened to them, and the potential cost the treatment would bring to them. According to two of the women, sometimes, they would think about the situation so much that they could not hear others when they were speaking with them.

*“I kept thinking*, *about the disease*. *Why should a young lady like me have this disease*? *What am I going to do now*? *Can I even pay*? *Sometimes*, *I do not even hear when people call my name”.*

(Participant 1, 34 years).

During treatment, the women also experienced psychological disruption. The women had bad feelings and cried. They were scared of the treatment methods, the potential results and side effects. One woman mentioned that when she was taken to the theatre and she saw the numerous knifes and machines that were going to be used on her, she became sad and begun to cry. Some of the women also saw the CT scan as a coffin and this traumatised them and increased their fear of death.

*“*. *I was made to sleep under a machine*. *When I got there*, *it was scary*, *it was like a coffin.”*

(Participant 8, 62 years).

The women experienced psychological disruption even after treatment. The women were afraid that the disease might recur. According to four of the women, they prayed constantly that the pain and troubles they had experienced should not recur. In summary, the patients experienced negative emotions and experiences at four levels, namely, symptoms, diagnosis, treatment and post treatment. To the women, cervical cancer is a disease that has a never-ending psychological disruption once it commences.

#### Economic disruption

The cervical cancer patients also experienced economic disruption in terms of their inability to work, reduction in income, medical bills, loss of asset and indebtedness. They had to go to hospital and could not trust others to handle their businesses, leading to their collapse. For instance, two respondents reported that they could not harvest from their farm because they were on admission, leading to losses. In all, most of the women (n = 9) lost their businesses:

*“All my work collapsed because I cannot do it on my own*. *Now*, *I can no longer work actively and my ability to walk is also not as fast as it used to be.”*

(Participant 15, 60 years).

As a result of their inability to work actively as they used to, their income stream was disrupted; yet, they had to pay healthcare bills. The women reported spending between GHC 300.00 (75 USD) and GHC 10, 000.00 (2500 USD) for treatment. Due to the expensive healthcare bill three women sold their assets and ten women took loans. For those who sold their assets they complained of losing value as they sold for less than the cost price of the asset they have in their possession:

*“I was so sick I could not work*, *but I needed money to pay my bills*. *So I sold my land*. *It was a difficult decision but I had to take it*. *Unfortunately*, *the buyer noticed that I needed the money so he offered me less than the market price.”*

(Participant 1, 34 years).

About five of the women took loans to finance their healthcare. The women borrowed between GHC100.00 (25 USD) and GHC6, 000.00 (1500 USD). They took loans from both formal (banks and microfinance) and informal source (friends and relatives). For those who took loans from microfinance institutions, they had to pay about 40 per cent interest on the loans they took. Some women lost their jobs and/or had a reduction in income; yet, had expensive medical bills to pay. Some respondents sold their assets and took loans with huge servicing and became indebted to the bank.

#### Social disruption due to cervical cancer

Break-up of social relationships was one of the disruptions experienced by cervical cancer patients. Some of the women explained that their partners deserted them and one of the women also had a disrupted relationship with her brother. Some of them reported that their partners left them because they could no longer endure having sex with them. The women said they felt disappointed without the emotional and the financial of their partners during their treatment. A woman also reported strained relationship with her brother because of the disease. The brother felt that he was spending too much money on her and therefore decided to abandon her.

*“Because of the disease*, *my husband went to marry another woman and left me.”*

(Participant 4, 44 years).

*“None of my family members supported me*. *Even my own sibling who comes after me did not mind me anymore*. *He said he had spent five times already so he would not spend money to take care of me again because my mother and my father did not leave him any inheritance to fall on*”

(Participant 11, 55 years).

## Discussion

The study found that most of the women did not know about the disease before and even after diagnosis. They lacked knowledge on the causes, risk factors, prevention methods and treatment options. The low level of cervical cancer knowledge among women has been reported in a number of other studies. Studies such as Francis et al. [19], Ndikom and Ofi [20], Abiodun et al. [21], and Williams [22] have also reported similar findings. They found little or no awareness about the disease among patients before they were diagnosed. This limited knowledge about the cervical cancer disease may be as a result of low or lack of intensive education or sensitisation on the disease among women in Ghana [8]. Consequently, the low level of knowledge may lead to low uptake of screening services [21]. This ‘health seeking behaviour’ may result in late identification of the disease and may delay in consulting orthodox health practitioners for treatment. Illness notification was the first experience women had of cervical cancer. The women experienced persistent bleeding, bodily pains, bodily discomfort and vaginal discharge. Some of the respondents experienced multiple of these bodily symptoms. Since most of the women did not know about the cervical cancer before they were infected, most of them used the changes they experienced in their body to detect that something was wrong with them. This is consistent with other studies that have suggested that a subjective experience through the process of ‘body-listening’ shaped disease notification and experience [23]. The women’s experience could be described as biographical disruption as a result of the disease. The experiences of the women could be viewed in terms of physical, psychological, economic and social disruption as a result of the disease. Chronic illnesses have been considered as an entry into a new social arena [13].

The initiation into the cervical cancer encounter could explain the disruption the women experienced. Physical disruption was experienced by the patients through pains in numerous parts of the body including the abdomen, waist and vagina. The inability to move and engage in their usual daily activities was also of concern to the patients. The patients also experienced psychological disruption because they were in critical situation of great uncertainty. This was compounded by the stigma attached to the disease and their ignorance of the state they find themselves, which led to the fear and hiding their disease status. A previous study found similar experiences among persons living with diabetes [24]. Also, the uncertain knowledge about the impact and cause of the disease as well as appropriate behaviour in the face of complications have been reported to cause stress among persons living with chronic diseases [25,26].

The disease impacted psychologically and economically on the patients. There was break down in economic activities because patients could not perform their businesses due to the ill health. Cost of treatment of the disease also rendered some patients indebted while others lost some assets due to attempts to pay for the treatment of the disease. The poverty of the women who are affected worsened because they are the breadwinners of the rural communities. This may have negative social and psychological implications leading to delay in the healing process.

The women also experienced social disruptions, which could be explained by the intolerance of family and friends in such a situation. The loss of social as well as intimate relationship was also reported among some patients. Other studies have reported similar findings about breakup of social relationships as a result of the disease [26–28]. It was also revealed that some of these women suffered social disruption due to their inability to engage in sexual intercourse with their partners. The disruption in the social lives of the cervical cancer patients was a source of constant stress, isolation, and low self-esteem.

One notable limitation of this study is that, it focused on only one health facility and therefore may not be able to establish the experiences of patients from other health facilities in the country. However, our findings are consistent with the concept of chronic disease as a biographical disruption suggesting that cervical patients may have similar experiences regardless of where they are located. Furthermore, the goal of the study was to unearth the real life experiences of cervical cancer patients in a rural setting rather than generalization.

## Conclusion

Patients had inadequate knowledge about cervical cancer, its symptoms, risk factors, treatment and prevention prior to the diagnosis of the disease. Cervical cancer caused physical, psychological, economic and social disruptions in the lives of its patients. Our findings contribute to the scanty literature on the experiences of cervical cancer patients, particularly in low resource settings. This study further provides vital insight into understanding the realities cervical cancer patients face on daily basis. This is critical for health promotion and improvement in the quality of life for people living with cervical cancer in low resource settings. Health education about the disease by health experts through the mass media can be a sure way of creating adequate awareness about the disease. Education programmes could focus on causes, risk factors, symptoms and screening services. Emphasis can be placed on preventive behaviours such as screening since it has the most positive impact on early detection and treatment. There should also be improved and sustained counselling sessions for patients as part of the treatment in order to enhance their coping ability. The counselling sessions should be geared towards addressing the various disruptions, which might arise from contracting the disease.

## Supporting information

S1 File Interview guide. (DOCX)
