# Supplementary material for: Experiences of cervical cancer patients in rural Ghana: An exploratory study
Source: PLoS One. 2017 Oct 11;12(10):e0185829. doi: 10.1371/journal.pone.0185829 (PMC5636100; doi:10.1371/journal.pone.0185829)
Supplement: S1 File — (DOCX) [file pone.0185829.s001.docx]

**Title:** **Experiences of Cervical Cancer Patients in Rural Ghana: An Exploratory Study**

**Interview Guide for Cervical Cancer Patients**

1. When did you first notice something was wrong with you?
2. Describe how you were feeling?
3. What did you do about it?
4. Whom did you talk to when you suspected something was wrong and why did you tell that person?
5. When did you take the decision to go to the hospital?
6. Who asked you to do the test? What was the reason behind your visit?
7. Who performed the test and where was it done?
8. When did you get to know that what you were suffering from is cervical cancer?
9. Who broke the news to you about your condition?
10. How was the news delivered to you?
11. How did you receive the news?
12. Let us discuss what you are suffering from?
13. Before you got to know of your condition, what other cancer diseases were you aware of?
14. Tell us about your sexual life - age of first sexual intercourse, with or without protection, number of partners (practice of sex with or without protection)

**Screening**

1. How was your experience with the screening?
2. What actually was done during the screening?
3. For how long did you have to wait to be screened**?**
4. How long did the screening last?
5. How long did it take for you to get the results?
6. What where you required to buy or provide for the screening?
7. How much did you pay for the screening?
8. How much would you have wished to be charged?
9. What kind of support did you receive from – family, friends, work, church, mosque etc.?
10. What influenced you to accept the screening – How, why, when?

**Diagnosis**

1. What were you told about your cancer?
2. Type and stage of cancer
3. Did you have additional tests/procedures?
4. What were you told about these?
5. How is the result of this test affecting you?

**Treatment**

1. What type of treatment were you given?
2. Describe your experience when you were put on treatment?
3. What influenced your decision to uptake the treatment- Faith etc.?
4. What sort of information were you giving concerning cancer/ treatment options?

**Ending**

Now lets us talk about your experience since you became aware that you have cervical cancer

1. Who kinds of support have you been receiving so far-financial, washing, cooking, cleaning, assistance with shopping, conversation with friends?
2. Who have been supporting you - husband, children, friends, church mosque etc.?
3. What pieces of advice have you been given?
4. What other experience would you like to share with other women to avoid cervical cancer?
5. What would you want to see changed in the way screening, diagnoses and treatment are done in Ghana?
6. What would you like the government to do in order to promote women uptake of cervical cancer screening in Ghana?
